# Elongin B promotes breast cancer progression by ubiquitinating tumor suppressor p14/ARF

**DOI:** 10.1007/s10565-024-09864-7

**Published:** 2024-04-23

**Authors:** Xin-Yi Sui, Xiao-Yan Ma, Yujin Hou, Shuo-Wen Cao, Zhi-Qing Wang, Li-Jun Jia, Lei Fan, Zhi-Ming Shao, Wen-Juan Zhang

**Affiliations:** 1 Department of Breast Surgery, Fudan University Shanghai Cancer Center, 270 Dong-An Road, Shanghai, 200032 P.R. China; 2 Key Laboratory of Breast Cancer in Shanghai, Department of Oncology, Shanghai Medical College, Fudan University, Shanghai, China; 3 Department of Oncology, General Hospital of Ningxia Medical University, Yinchuan, China; 4 Cancer Institute, Longhua Hospital, Shanghai University of Traditional Chinese Medicine, Shanghai, China

**Keywords:** ELOB, p14/ARF, Ubiquitination, Breast cancer, Anti-cancer target

## Abstract

**Abstract:**

Elongin B (ELOB), a pivotal element in the ELOB/c-Cullin2/5-SOCS-box E3 ubiquitin-protein ligase complex, plays a significant role in catalyzing the ubiquitination and subsequent degradation of a broad spectrum of target proteins. Notably, it is documented to facilitate these processes. However, the regulatory role of ELOB in breast cancer remains ambiguous. In this study, through bio-informatic analysis of The Cancer Genome Atlas and Fudan University Shanghai Cancer Center database, we demonstrated that ELOB was over-expressed in breast cancer tissues and was related to unfavorable prognosis. Additionally, pathway enrichment analysis illustrated that high expression of ELOB was associated with multiple cancer promoting pathways, like cell cycle, DNA replication, proteasome and PI3K − Akt signaling pathway, indicating ELOB as a potential anticancer target. Then, we confirmed that both in vivo and in vitro, the proliferation of breast cancer cells could be significantly suppressed by the down-regulation of ELOB. Mechanically, immunoprecipitation and in vivo ubiquitination assays prompted that, as the core element of Cullin2-RBX1-ELOB E3 ligase (CRL2) complex, ELOB regulated the ubiquitination and the subsequent degradation of oncoprotein p14/ARF. Moreover, the anticancer efficacy of erasing ELOB could be rescued by simultaneous knockdown of p14/ARF. Finally, through analyzing breast cancer tissue microarrays and western blot of patient samples, we demonstrated that the expression of ELOB in tumor tissues was elevated in compared to adjacent normal tissues. In conclusion, ELOB is identified to be a promising innovative target for the drug development of breast cancer by promoting the ubiquitination and degradation of oncoprotein p14/ARF.

**Graphical headlights:**

ELOB is highly expressed in breast cancer.

High ELOB levels were positively associated with poor prognosis.

ELOB promotes p14/ARF degradation as part of the Cullin2-RBX1-ELOB E3 ligase complex.

ELOB is a promising biomarker for breast cancer.

**Graphical Abstract:**

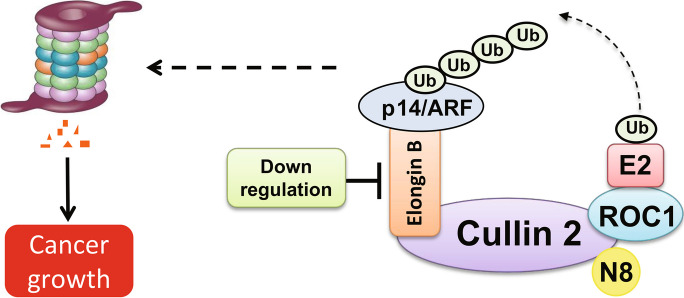

**Supplementary Information:**

The online version contains supplementary material available at 10.1007/s10565-024-09864-7.

## Introduction

Breast cancer reigns as the predominant malignancy afflicting women globally, asserting its prominence as the most prevalent tumor in the female population worldwide (Fan et al. 2014; Hutchinson 2010; Woolston 2015). Research findings have hinted at the potential involvement of ubiquitination modifications in both the onset and advancement of cancer (Ding et al. 2021; Li et al. 2023). The ubiquitin proteasome system (UPS) stands as a cornerstone pathway in eukaryotic cells, pivotal in orchestrating the intricate orchestration of protein degradation to maintain cellular equilibrium and functionality. By targeting specific proteins for degradation via ubiquitination and subsequent proteasomal processing, the UPS ensures the timely removal of damaged or surplus proteins, thereby influencing various cellular processes and signaling pathways (Ciechanover 1994). Ubiquitination, a complex post-translational modification process, is characterized by the covalent linkage of ubiquitin molecules to designated lysine sites on target proteins, thereby regulating a vast array of cellular signaling networks crucial for maintaining protein balance and cellular homeostasis (Pickart 2001). The process of attaching ubiquitin to substrates is a sequential, multi-phase event that includes the participation of ubiquitin-activating enzyme (E1), ubiquitin-conjugating enzyme (E2), and ubiquitin ligase (E3). Initially, E1 catalyzes the formation of a thioester linkage, binding ubiquitin to its active site. This activated ubiquitin is then transferred to E2 through a similar thioester linkage. E3 ligases work in tandem with E2 to mediate the attachment of ubiquitin to the designated substrate (Eldridge and O’Brien 2010; Park et al. 2020). E3 ubiquitin ligases can be classified into three categories based on the presence or absence of HECT (homologousto the E6-AP carboxyl terminus), RING (really interesting new gene), and U-box structural domains (Ardley and Robinson 2005). The Cullin-RING (CRL) E3 ubiquitin ligase plays a pivotal role in the ubiquitin–proteasome system, orchestrating the targeted degradation of specific proteins through accurate ubiquitination tagging. Remarkably, about 20% of proteins undergo degradation through this pathway. The CRL E3 ubiquitin ligase targets a wide array of substrates, encompassing proteins critical for cell cycle regulation, signal transduction pathways, transcription factors, and proteins with oncogenic potential (Petroski and Deshaies 2005). Therefore, under normal physiological conditions, CRL E3 ubiquitin ligase regulates various biological processes, including the cell cycle, cell proliferation and differentiation (Chairatvit and Ngamkitidechakul 2007; Petroski and Deshaies 2005; Salon et al. 2007). When its activity is dysregulated, it can contribute to the development of many diseases, including tumors.

Elongin B (ELOB) is a 118 amino acid protein (Aso et al. 1995) that was initially identified as a regulatory subunit of RNA polymerase II elongation (Aso et al. 1995). Together with ELOA (transcriptionally active component) and ELOC (another regulatory subunit), ELOB forms the Elongin (SIII) complex, which plays a role in regulates the elongation process of RNA polymerase II by preventing transient pausing of the polymerase at various sites within the transcriptional unit. Moreover, ELOB has been found to be a part of several UPSs, including CRL2 and CRL5. Specifically, CRL2 consists of RBX1, Cullin-2, connexin ELOB/C and substrate recognition proteins (Nguyen et al. 2015). Von Hippel-Lindau (VHL) protein is a well-studied recognition protein that interacts with CLR2. VHL protein inhibits vasculature by connecting CRL2 and hypoxia-inducible factor-α (HIF1α), thereby suppressing angiogenesis and cell proliferation (Maxwell et al. 1999; Ohh et al. 2000). In VHL syndrome, mutations in VHL impede its association with ELOB/C, leading to the inactivation of the CRL2 E3 ligase complex. This disruption causes an accumulation of HIF1α (Hudler and Urbancic 2022).

The classical oncoprotein ARF derived from the Ink4a/ARF (CDKN2A) gene, assumes the aliases p14/ARF in human cellular contexts and p19/ARF in murine cellular environments, underscoring its pivotal role in the intricate regulatory networks governing cell cycle progression and tumor suppression mechanisms (Quelle et al. 1995). Numerous previous studies have demonstrated that ARF exert a tumor-suppressive function by activating the p53 downstream pathways through inhibiting the ubiquitin ligase MDM2 and impeding the ARF-BP1/Mule-mediated p53 degradation process (Chen et al. 2005; Sherr 2006; Zindy et al. 1998). Additionally, ARF can act as a tumor suppressor through p53-independent pathways. ARF can suppress cell growth by interacting with nucleophosmin1 (NPM1) to regulate ribosomal RNA transcription and processing, or by modulating VEGFA mRNA translation to prevent angiogenesis (Itahana et al. 2003; Lee et al. 2005). Furthermore, alterations in p14/ARF expression level in breast cancer suggest that it might be a potent marker of breast cancer progression (Pare et al. 2016). Retrospective analyses have revealed that a sequential increase in p14/ARF expression from normal to atypical ductal hyperplasia to ductal carcinoma in situ, with p14/ARF expression stabilizing as the tumor progresses. As the tumor begins to proliferate, p14/ARF expression gradually increases as a natural response to tumor suppressor genes counteracting growth. However, when the lesion evolves to be invasive, the p14/ARF expression tends to remain at a lower level. In summary, p14/ARF may regulate the inhibition of tumor cell proliferation through multiple pathways.

Here, we report that ELOB, identified as a component of CRL2 that promotes degradation of the substrate p14/ARF, was detected to be markedly overexpressed, and was related to unfavorable prognosis in breast cancer. Targeting ELOB prevented p14/ARF degradation and inhibited cancer cell proliferation. All in all, our study indicates a novel oncogenic target of breast cancer, ELOB, and targeting ELOB could prevent breast cancer proliferation by suppressing the breakdown of p14/ARF.

## Materials and methods

### Bioinformatics analysis

We obtained RNA-seq data with clinical information from The Cancer Genome Atlas (TCGA, https://tcga-data.nci.nih.gov/tcga/), cBioPortal (https://www.cbioportal.org/) (Cerami et al. 2012; Kao et al. 2021; Lamb et al. 2006) and Fudan University Shanghai Cancer Center (FUSCC) database (Jiang et al. 2019). Additionally, scRNA-seq data for five TNBC patients were from the Gene Expression Omnibus (GEO, https://www.ncbi.nlm.nih.gov/geo/) database ID GSE148673. Then, we utilized the Kaplan–Meier method to generate survival curves, followed by an evaluation using the log-rank test. Additionally, employing the Kaplan–Meier plotter online, we analyzed the relationship between ELOB expression and prognosis (https://kmplot.com/analysis/). Methsurv database is used for the analysis of DNA methylation (https://biit.cs.ut.ee/methsurv/) (Anuraga et al. 2021; Wang et al. 2020; Xing et al. 2021). Differential analysis was performed using the limma package in the R programming environment. Genes that met the filtering criteria of P < 0.05 and |log2 fold change (FC)|> 0.58 were classified as differentially expressed genes. For a deeper exploration of the biological significance of these genes, we performed Gene Ontology (GO) enrichment and Kyoto Encyclopedia of Genes and Genomes (KEGG) pathway analyses using the R package "clusterProfiler". Additionally, we applied Gene Set Enrichment Analysis (GSEA) to assess the potential impact of ELOB gene expression on pathway activity. Furthermore, for the single cell analysis, we utilized the "Seurat" software package and the t-distributed Stochastic Neighbor Embedding (tSNE) method for non-linear dimensionality reduction and visualization. Subsequently, cell clusters were identified and annotated using the "singleR" software package and established labeling methods. Connectivity Map (CMap) an online analysis site that can be used to discover the mechanism of action of small molecules and to inform clinical trials (https://clue.io) (Lamb et al. 2006; Subramanian et al. 2017; Wang et al. 2020).

### Cell lines and cell culture

The human breast cancer cell lines MDA-MB-231, BT-549, and T-47D were obtained from the American Type Culture Collection, while the human embryonic kidney cell line HEK293T was kindly donated by Professor Guohong Hu in 2014. Each cell line underwent thorough authentication and testing to verify their freedom from mycoplasma contamination. T-47D and BT-549 cells were grown in RPMI1640 medium enriched with insulin, and MDA-MB-231 cells thrived in DMEM medium. The culture media were fortified with fetal bovine serum and penicillin–streptomycin. All cells were cultured in a 5% CO_2_ incubator at 37°C. To maintain their quality and reliability, the cell lines were utilized within six passages after procurement.

### Plasmid construction

Cell transfection was conducted using siRNA from Genepharma (China). The siRNA sequences were as listed below: ELOB,5′-UGAACAAGCCGUGCAGUGA-3′, 5′-AGCGGCUGUACAAGGAUGA-3′; RBX1, 5′-GACTTTCCCTGCTGTTACCTAATT-3′, 5′-CTGTGCCATCTGCAGGAACCACATT-3′; Cullin2, 5′-GCACAAUGCCCUUAUUCAA-3′, 5′-GCAGAAAGACACACCACAA-3′. Besides, to obtain the overexpression plasmids, the coding regions of ELOB and p14/ARF were tagged with FLAG and HA, respectively, and cloned into the empty loadling plasmids. Overexpression plasmids for ELOB and p14/ARF were designed and synthesised by GeneChem Corporation (Shanghai, China).

### Western blot (WB) analysis

In brief, cell lysis was carried out by Pierce T-PER® Tissue Protein Extraction Reagent (Thermo Fisher Scientific Inc., USA, catalog numbers B14001, B15001A + B), enriched with protease and phosphatase inhibitors (Bimake, USA, catalog numbers B14001, B15001A + B). The resultant lysate underwent centrifugation at 12,000 rpm for 15 min, leading to the collection of the supernatant. Protein concentration was determined using the Bicinchoninic Acid (BCA) Protein Assay Kit (Solarbio, catalog number PC0020). Subsequently, 20–30 μg of proteins were separated via SDS-PAGE and transferred onto PVDF membranes (Millipore, USA, catalog numbers IPVH00010, ISEQ00010). The antibody targeting p14/ARF (AP51072) was procured from ABGENT, China. Additionally, ELOB antibody (ab151743) and p14/ARF antibody (ab11048) were acquired from Abcam.

### Coimmunoprecipitation

Cells cultivated in 10-cm dishes underwent a single wash with 1 × PBS and were subsequently lysed by pre-chilled NP-40 lysis buffer, which consisted of 50 mM Tris–HCl at pH 7.5, 150 mM NaCl, and 0.5% NP-40. To this lysis buffer, 1 mM PMSF was added. Following a 5-min centrifugation at 12,000 g and 4°C, we discarded the precipitate filled with cellular debris, collecting the clear supernatant for use in Co-IP experiments. In a nutshell, the cell extracts were subjected to an incubation with the primary antibody ELOB (ab151743, Abcam) or a control IgG in a rotary incubator overnight at 4°C. Subsequently, the sample underwent a further two-hour incubation with protein A/G magnetic beads (Bimake). The immunoprecipitates were then subjected to three washes and used for subsequent immunoblot analysis.

### Immunoblotting and cycloheximide (CHX)-chase assay

Cell lysates were generated and analyzed via immunoblotting. We used antibodies against RBX1 (ab133565), Cullin2 (ab166917), and ELOB (ab151743), all sourced from Abcam. During the cycloheximide (CHX) chase assays, the cells were treated with 50 μg/mL CHX (C4859, Sigma, Germany), alongside either 1.0 μM/L MLN4924 or DMSO, for predetermined periods.

## Immunofluorescence and confocal microscopy

After fixation in 0.4% paraformaldehyde, the cells were permeabilized with 0.5% Triton X-100, blocked with 1% BSA-PBS, and then incubated overnight at 4°C with primary antibodies targeting FLAG (F1804, Sigma) and anti-HA (H6908, Sigma). Subsequently, staining with secondary antibodies (10400C, 10500C, Invitrogen) was carried out for 1 h. This was followed by staining with 4′,6-diamidino-2-phenylindole (DAPI, Sigma) and sealing to prevent quenching. Finally, fluorescence images were obtained through the utilization of a confocal scanning microscope (LSM880 with Fast Airyscan, Zeiss).

### *In vivo ubiquitination assay*

For the assessment of endogenous p14/ARF ubiquitination, MDA-MB-231 cells were transfected with siRNA oligonucleotides aimed at ELOB, in addition to a control siRNA with a scrambled sequence. Ninety-six hours following transfection, the cells received a 2-h MG-132 treatment. Lysis of the cells was achieved using 100 μL of a 1% SDS-containing cell lysis buffer. Post boiling of the lysates for 10-min, they were diluted in a tenfold volume of an SDS-free lysis buffer, then proceeded to immunoprecipitation using an anti-p14/ARF antibody (ab3642, Abcam), and immunoblot analysis employing an anti-ubiquitin antibody (sc8017, Santa Cruz, USA).

### Immunohistochemical staining of human tumor tissue array

Aiyou Biotechnology, located in Shanghai, China, conducted Immunohistochemistry (IHC) staining on tissue arrays. The ELOB antibody (sc-133090, Santa Cruz) was employed for this purpose. The tissue array sections, which had a thickness of 5 microns, underwent dehydration and subsequent peroxidase blocking. Following this, the primary antibody was applied and allowed to incubate for 30-min using the DAKO AutoStainer. To facilitate detection, the DakoCytomation EnVision + System-HRP detection kit from Dakocytomation was utilized. Hematoxylin was used for counterstaining the slides. The stained slides were then subjected to microscopic observation and image capture. The Immunohistochemistry results were further evaluated using the H-score (or “histo” score), a semi-quantitative method that ranges from 0 to 300 (Hirsch et al. 2003; Pirker et al. 2012). Two scientists independently conducted IHC assessments, and samples that were not paired were excluded.

### Tumor tissue collection and ethical statement

Immunoblotting was performed at FUSCC using twenty pairs of breast and breast cancer tissues sourced from patients. In line with ethical standards, all enrolled patients granted written informed consent before the study, following the Declaration of Helsinki to authorize the scientific research utilization of tissues and data. Additionally, the Research Ethics Committee of the FUSCC, provided approval for this study.

### Generation of stable cell lines

To achieve ELOB knockdown, we used ELOB sgRNA oligos. The forward oligo sequence of sgRNA#1 is 5′-CACCGCGTGATTACAGCCCCCAGCG-3′, and the reverse oligo sequence of sgRNA#1 is 5′-AAACCGCTGGGGGCTGTAATCACGC-3. The forward oligo sequence of sgRNA#6 is 5′-CACCGCGAACTGAAGCGCATCGTCG-3′, and the reverse oligo sequence of sgRNA#6 is 5′-AAACCGACGATGCGCTTCAGTTCGC-3. To achieve P14/ARF knockdown, we used p14/ARF sgRNA oligos. The forward oligo sequence, 5′-CACCGTCTTGGTGACCCTCCGGATT-3′, and the reverse oligo sequence, 5′-AAACAATCCGGAGGGTCACCAAGAC-3′, were used. These oligos were inserted into the LentiCRISPR v2 plasmid (Plasmid #52,961, Addgene, USA). For transfection, HEK293T cells were transfected with Lipofectamine 2000, introducing a total of 4.0 μg of LentiCRISPR plasmid with SgRNA, 3.0 μg of packaging plasmids psPAX2, and 1.0 μg of pMD2.G. Following a 36-h incubation period, the virus supernatant was harvested and combined with polybrene to boost infection efficacy. Infected cells were then isolated through exposure to 2 μg/mL puromycin over a two-week period or by employing fluorescence-activated cell sorting (FACS).

### Cell viability assay and clonogenic survival assay

The initial cell distribution in the 96-well plates was set at a density of 3 × 10^3^ cells per well. To assess proliferation, the ATPLite Luminescence Assay kit (PerkinElmer, USA) was employed following the manufacturer’s protocol. Five distinct experiments were conducted for this analysis. In the clonogenic assay, approximately 200 MDA-MB-231 cells were introduced into 6-well plates and subsequently incubated for a period of 10 days. The quantification of colonies, defined as containing 50 or more cells, was executed by an inverted microscope. The quantification of colonies, defined as those consisting of 50 cells or more, was carried out using an inverted microscope. To ensure the reliability of the results, three independent experiments were conducted.

### Subcutaneous xenograft in mice

A total of four separate cohorts were formed by randomly dividing female BALB/c nude mice, aged between 4 and 6 weeks. The treatment procedures strictly adhered to established guidelines. The research protocol underwent meticulous scrutiny and received approval from an internal committee responsible for reviewing animal protocols. Tumor xenograft sizes were measured tri-weekly, with the volume calculated by the formula: (length x width^2^) / 2. Researchers were kept blind to group allocations, ensuring impartiality during both the experimental process and the analysis of outcomes. Upon euthanization of the mice, the subcutaneous tumors were excised for further immunoblotting studies.

### Statistical analysis

To evaluate the statistical significance of group differences, we utilized GraphPad Prism 6 and R software version 4.2.2. The student t-test was applied to compare parameters among the groups. The statistical methods of Log-rank test and Cox regression were employed for survival analysis. Data were displayed as mean ± standard deviation.

## Results

### Targeting the overexpression of ELOB in breast cancer resulted in suppressed tumor growth

**Fig. 1:**
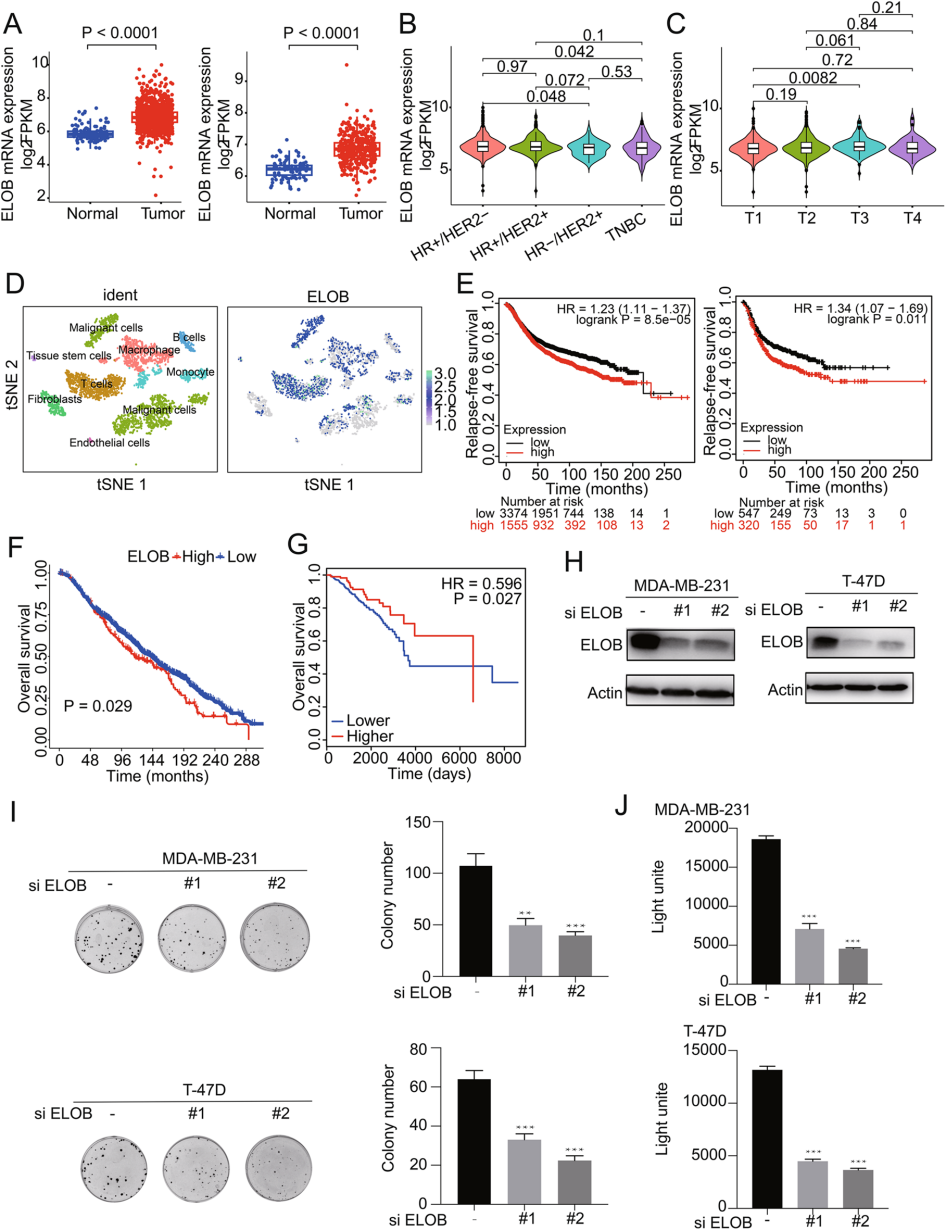
ELOB is overexpressed in breast cancer. **A** ELOB expression levels in normal tissue and tumor samples from TCGA (left, n = 1217) and FUSCC (right, n = 448) database in breast cancer and TNBC, respectively. **B** ELOB expression in breast cancer with different subtype. HR + /HER2- patients had higher expression. **C** ELOB expression in breast cancer with different tumor size. Patients with T3 had higher ELOB expression. **D** Single-cell RNA-seq demonstrates ELOB expression in tumor cells and other cells. ELOB expression was higher among tumor cells. **E** ELOB overexpressions were associated with decreased relapse-free survival probability in all subtypes of breast cancer (left) and triple-negative breast cancer (right). Patients with higher ELOB expression had worse prognosis. Data obtained were from the dataset (200085_s_at and 200085_s_at) through a comprehensive search using Kaplan–Meier plotter. com.** F** Relationship between ELOB expression and overall survival. Patients with higher ELOB expression had shorter overall survival. **G** Relationship between ELOB promoter region methylation and overall survival. patients with lower methylation levels in the ELOB promoter region have a better prognosis. **H** Knockdown ELOB in MDA-MB-231 and T-47D cells and validation of knockdown efficiency. ** I** Clone formation assays verified that ELOB down-regulation inhibited cell proliferation in the MDA-MB-231 and T-47D cell lines. Knockdown of ELOB inhibits tumor proliferation. ** J**  ATPlite assay verified that ELOB down-regulation inhibited cell proliferation in the MDA-MB0231 and T-47D cell lines

To elucidate ELOB’s expression dynamics, we analyzed its mRNA levels in breast cancer tissues versus adjacent normal tissues using data from the TCGA and FUSCC databases. The result demonstrated that ELOB was over expressed in all molecular subtypes of breast cancer (Fig. 1A) with the highest expression of ELOB in HR + /HER2- subtypes and the lowest in TNBC (P < 0.05, Fig. 1B). Furthermore, a positive correlation was observed between ELOB expression and tumor size, with a significant increase from stages T1 to T3 (P < 0.05, Fig. 1C). To investigate in which cells ELOB was predominantly expressed, we performed a single-cell analysis of 8 cell populations from 5 breast cancer cases (Fig. 1D).The expression of ELOB was observed in tumor cells, T cells, and macrophages among which the highest expression level was observed in tumor cells (Fig. 1D). Moreover, survival analysis indicated that high ELOB expression was associated with worse relapse-free survival (RFS) in all subtypes of breast cancer (Fig. 1E). An analysis of the cBioportal database unveiled a relationship between ELOB mRNA expression and overall survival (OS), and patients with higher ELOB expression had a poorer prognosis (Fig. 1F). Analysis of DNA methylation through the Methsurv database indicated that patients exhibiting reduced methylation levels in the ELOB promoter region were associated with a comparatively improved prognosis (Fig. 1G). Thus, we hypothesized the up-regulated ELOB might regulate the malignant phenotype of breast cancer. To validate the aforementioned hypothesis, we utilized ELOB siRNA to down-regulate ELOB in MDA-MB-231 and T-47D cell lines (Fig. 1H). The clonogenic assay and cell viability assay revealed that cell proliferation was suppressed following ELOB knockdown, suggesting that ELOB may serve as a potential anticancer target in breast cancer (Fig. 1I, J).

### Bioinformatics analyses suggest ELOB is related to the malignant biological behavior

**Fig. 2:**
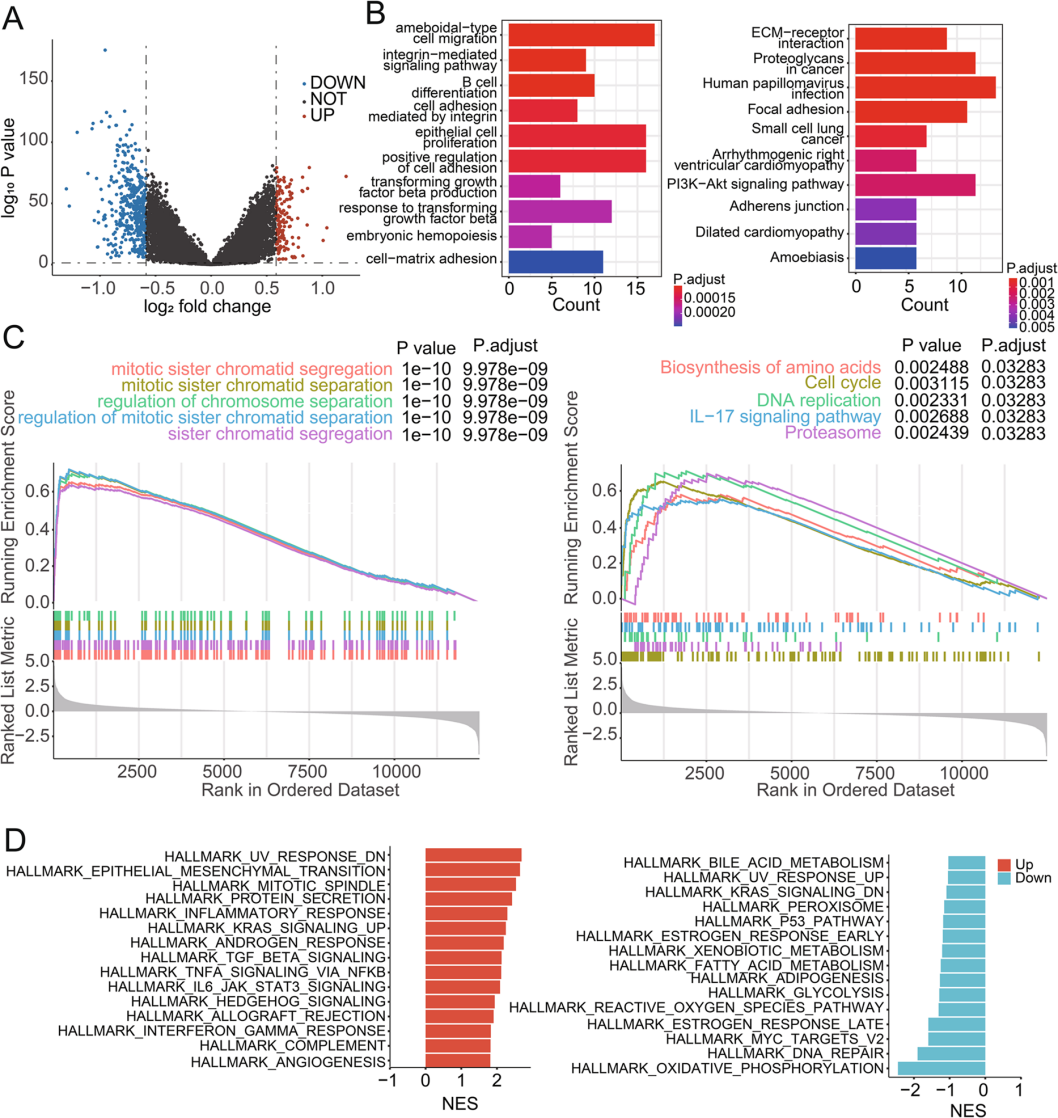
Pathway enrichment in ELOB high and low expression groups. **A** Different gene volcano was plotted according to the level of ELOB expression. **B** KEGG (left) and GO (right) enrichment analysis of different genes. **C** Enrichment plots from GSEA. D Enrichment analysis of the HALLMARK gene set

In order to further elucidate the bio-function of ELOB among patients with breast cancer, bioinformatics analyses of the TCGA database. First, patients were grouped according to the median ELOB expression in the TCGA database. Analysis of differential gene expression revealed 144 up-regulated genes and 421 down-regulated genes in the high ELOB expression group (P < 0.05, |log (FC)|≥ 0.58, Fig. 2A). GO enrichment analysis suggested that distinct genes were mainly enriched in the pathways such as epithelial cell proliferation and transforming growth factor beta production (Fig. 2B). KEGG enrichment analysis suggested that distinct genes were mainly enriched in the pathways such as ECM-receptor interaction, proteoglycan in cancer, and PI3K-Akt signaling pathway and so on (Fig. 2B). In addition, cell cycle, DNA replication, and prodeasome were active in the ELOB high expression group (Fig. 2C). Besides, the HALLMARK pathway GSEA discovered that epithelial mesenchymal transition, UV-responsive pathways, and KRAS signaling were enriched in the ELOB high-expression group (Fig. 2D). These results suggest that ELOB is strongly related to the malignant biological behaviors among patients with breast cancer.

#### ELOB regulates the degradation of p14/ARF through UPS

**Fig. 3:**
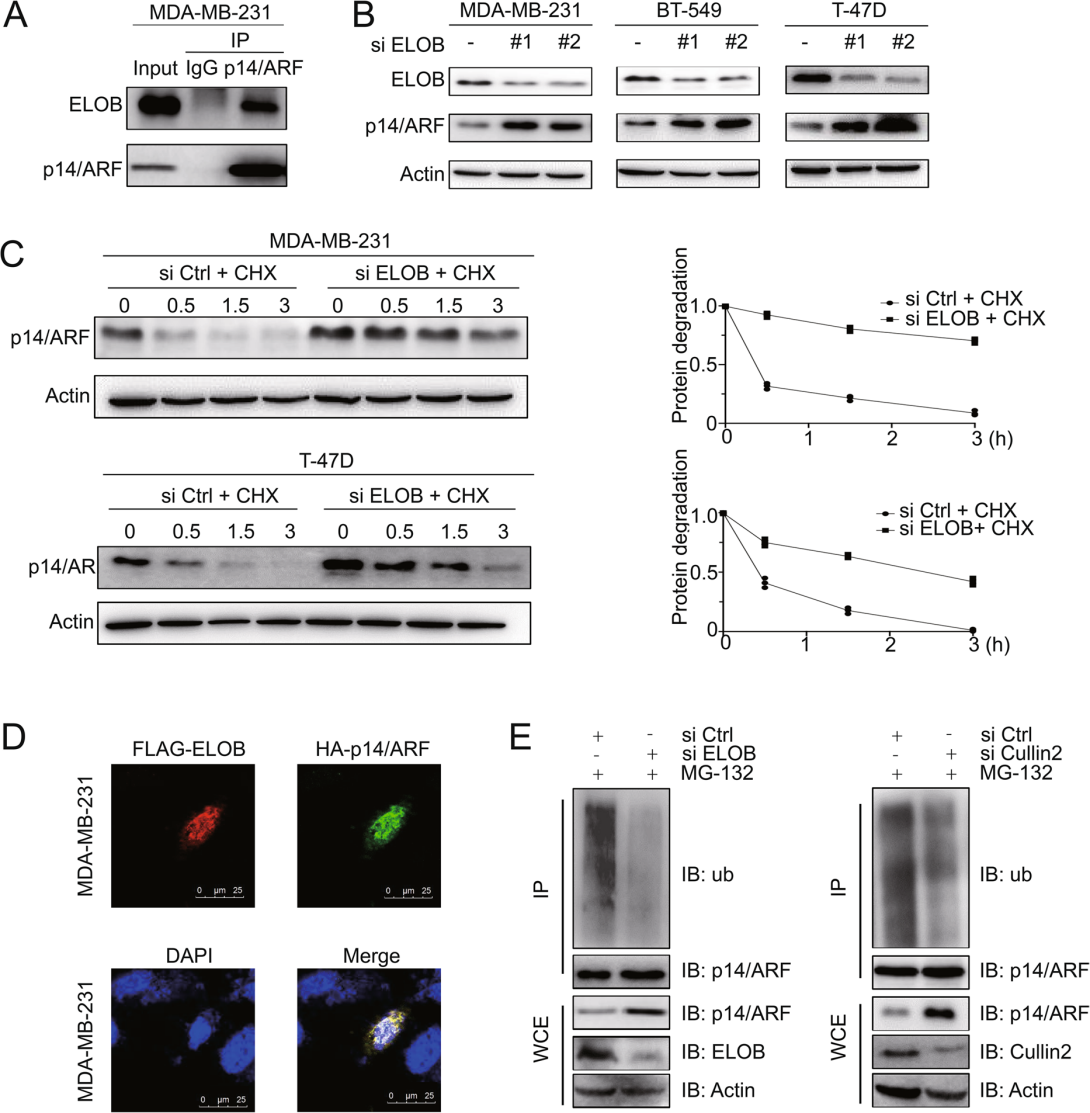
The Cullin2-RBX1-ELOB complex mediates p14/ARF degradation through ubiquitination. **A** ELOB interacts with p14/ARF at endogenous levels. MDA-MB-231 cells were pretreated with MG-132 for 2-h. Cells were harvested and subjected to immunoprecipitation with anti-ELOB Ab and immunoblotting with anti-p14/ARF Ab. **B** Downregulation of ELOB induces p14/ARF accumulation in MDA-MB-231, BT-549 and T-47D cells lines. Cells were transfected with control or ELOB siRNA for 96-h and subjected for immunoblotting analysis using antibodies against ELOB and p14/ARF with Actin as a loading control. **C** Downregulation of ELOB delays the degradation and extends the half-life of p14/ARF. MDA-MB-231 and T-47D cells were transfected with control or ELOB siRNA for 96-h and then treated with 50 μg/mL CHX for indicated time before subjected to immunoblotting analysis. **D** Immunofluorescence staining revealed the cellular location of exogenous FLAG-ELOB (red) and exogenous HA-p14/ARF (green). **E** Cullin2-RBX1-ELOB complex mediates polyubiquitination of p14/ARF in MDA-MB-231 cells. Cells were transfected with control or ELOB, Cullin2 siRNA respectively for 96-h, then treated with MG-132 for 2-h. Cells were extracted and subjected to immunoprecipitation with p14/ARF Ab and immunoblotting with anti-ub Ab. All data were representative of three independent experiments. Data represented means, and error bars were standard deviation

Next, we explored the downstream effector molecules of ELOB. It has been demonstrated that ELOB is a linker protein for E3 ligase and regulates the degradation of p14/ARF (Zhang et al. 2021). We hypothesized that ELOB regulates p14/ARF degradation through the CRL2 E3 ligase. In order to validate this hypothesis, the intracellular interaction between ELOB and p14/ARF was initially confirmed through an immunoprecipitation assay utilizing anti-ELOB antibodies and subsequent immunoblotting with anti-p14/ARF antibodies (Fig. 3A). Following this, ELOB was targeted for knockdown using siRNA to disrupt the CRL2 complex, and the levels of p14/ARF protein were evaluated via immunoblotting in various human breast cancer cell lines. Remarkably, the downregulation of ELOB led to a significant accumulation of p14/ARF protein across all breast cancer cell lines (Fig. 3B). Additionally, the impact of ELOB downregulation on the degradation of p14/ARF was investigated in the presence of CHX to inhibit protein translation. The results demonstrated that down-regulation of ELOB significantly delayed the degradation of p14/ARF and prolonged the protein half-life of p14/ARF (Fig. 3C). Immunofluorescence staining further confirmed the co-localisation of HA-p14/ARF and FLAG-ELOB (Fig. 3D). After determining that ELOB degraded p14/ARF via the ubiquitin–proteasome system, we explored other components of the CRL2 complex. It was observed that the knockdown of ELOB and Cullin2 through siRNA both suppressed the polyubiquitination of p14/ARF in cells (Fig. 3E). These findings confirm that ELOB together with RBX1 and Cullin2 constitutes an E3 ligase complex that targets p14/ARF and interacts with it, resulting in p14/ARF ubiquitination and degradation.

#### ELOB knockout inhibits cell proliferation by increasing p14/ARF

**Fig. 4:**
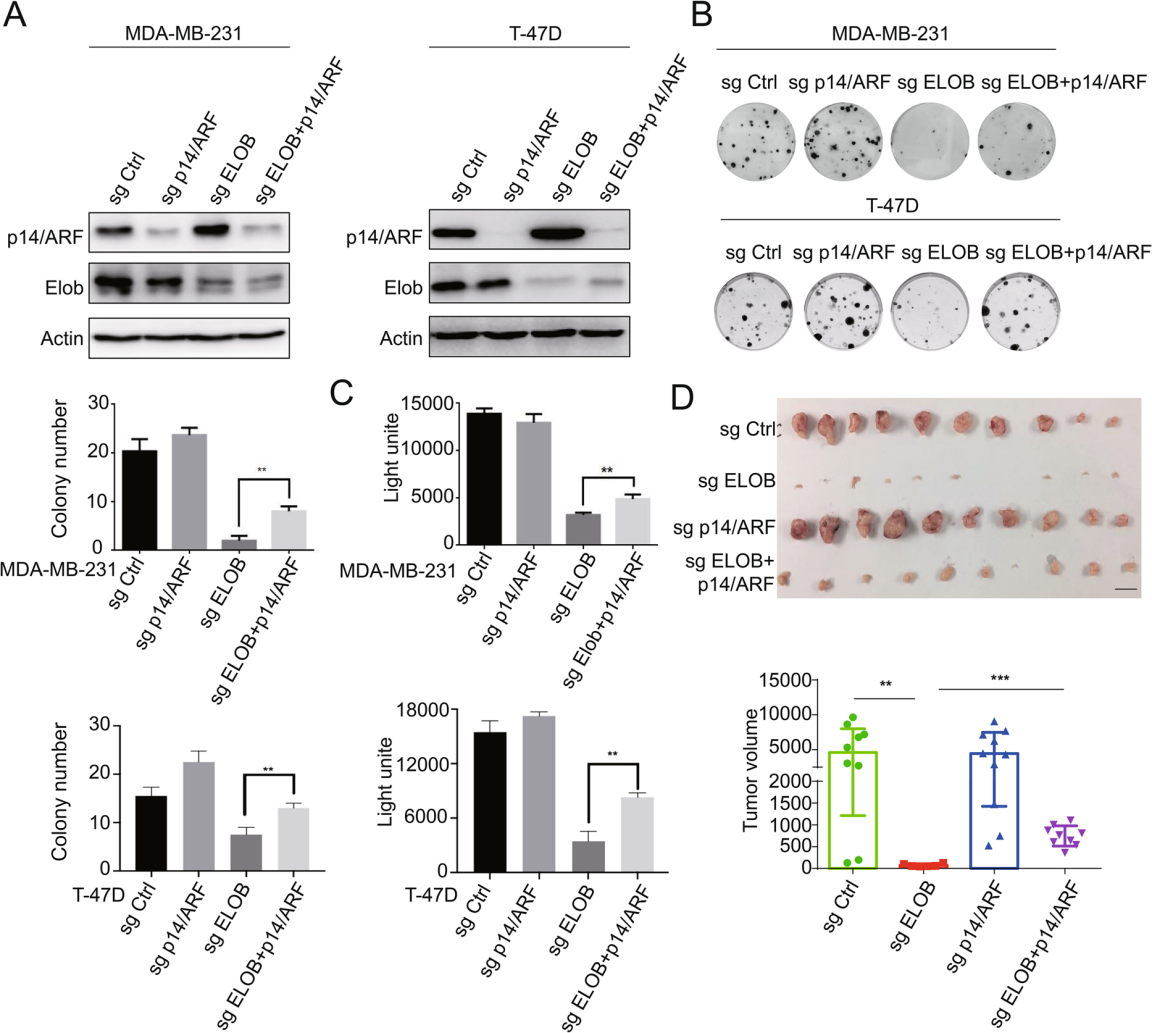
ELOB serves as a novel anticancer target through p14/ARF accumulation. **A** Stable MDA-MB-231 and T-47D cell lines with ELOB downregulation, p14/ARF downregulation, ELOB downregulation and p14/ARF downregulation were generated and their efficacy was demonstrated by immunoblotting. **B** Clone formation assay was performed using knockdown cells. Downregulation ELOB and p14/ARF can partially revert the inhibition of cell proliferation by downregulation ELOB alone. **C** ATPlite assay was performed using knockdown cells. **D** Nude mice were injected subcutaneously with ELOB downregulated, p14/ARFN downregulated, both ELOB downregulated and p14/ARF downregulated MDA-MB-231 cells (n = 10). Downregulation ELOB and p14/ARF can partially revert the inhibition of cell proliferation by downregulation ELOB alone in vivo. The tumor was collected immediately after the mice were euthanized. The figure shows representative tumor sizes of the 4 groups, scale bar: 10 mm

The elevated expression of ELOB in breast cancer suggests its potential as a promising target for anticancer therapy. To validate this hypothesis, we first constructed ELOB knock out MDA-MB-231 and T-47D cell lines (sgRNA#6), p14/AFR knockout MDA-MB-231 cell lines (sgRNA#1), both ELOB (sgRNA#6) and p14/AFR (sgRNA#1) knockout cell lines and the negative control cell lines (non-targeted sgRNA) based on CRISPER-Cas9 technology (Fig. 4A). The clonogenic assay and cell viability assay showed that cell proliferation was inhibited after ELOB knockout. This could be rescued by additional p14/ARF knockout (Fig. 4B, C). Next, we determined the effect of ELOB knockout in vivo. Four groups of cells described above were subcutaneously injected into nude mice to observe tumor growth (n = 10). Comparing with the negative control group, the tumor formation was almost repressed in the ELOB knockout group. Furthermore, the suppressed tumor growth resulting from ELOB knockout was partially restored by concomitant downregulation of p14/ARF, indicating that the antitumor impact of ELOB depletion may be linked to the accumulation of p14/ARF (Fig. 4D).

#### ELOB is highly expressed in human breast cancer tissue

**Fig. 5:**
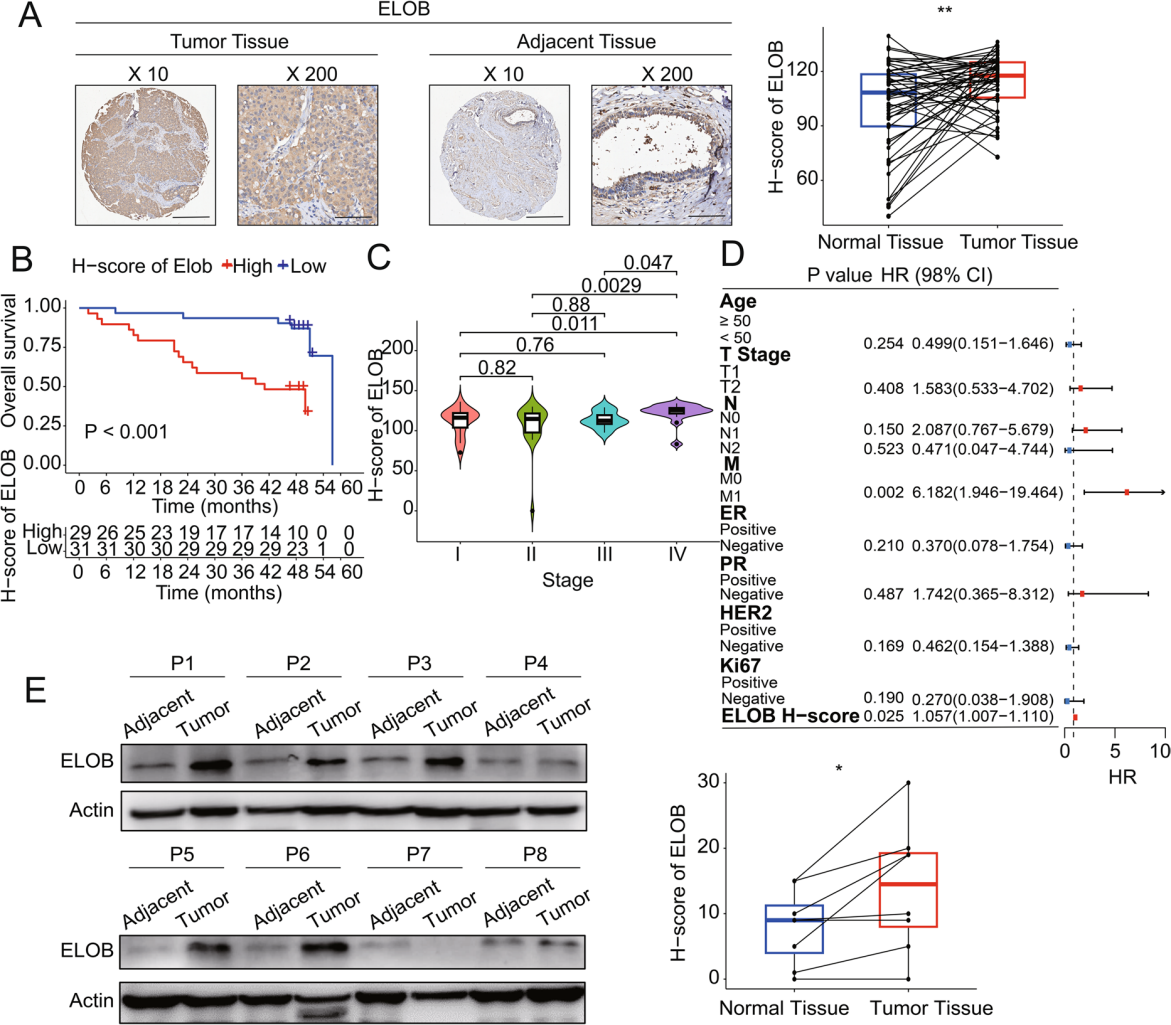
High ELOB expression is associated with poor prognosis among breast cancer patients. **A** Immunohistochemistry staining of ELOB protein in the normal breast tissues and breast cancer tissues. ELOB expression was higher in tumor tissues. **B** Relationship between ELOB H-score in Immunohistochemistry staining and overall survival. Patients with high ELOB expression had shorter overall survival. **C** Relationship between ELOB H-score in Immunohistochemistry staining and tumor stage. ELOB expression was higher in patients with stage IV. **D** Multivariate cox regression analyses for overall survival in Immunohistochemistry staining. **E** Western blot of ELOB expression in breast cancer tissues (n = 8) and adjacent normal tissues (n = 8). ELOB expression was higher in tumor tissues

ELOB expression was determined at protein levels though IHC staining in 61 tumor tissues and the paired adjacent normal tissues. Tumor tissues displayed elevated levels of ELOB expression in contrast to adjacent normal tissues. We followed up these patients and noted that ELOB overexpression was associated with shorter OS (Fig. 5B). In addition, the H-score of ELOB immunohistochemical staining was positively correlated with tumor staging (Fig. 5C). Notably, multivariate logistic regression analysis demonstrated that high levels of ELOB were independent predictors of OS (Fig. 5D). Following this, immunoblot analysis was performed on 20 matched pairs of breast cancer and adjacent normal tissues to evaluate ELOB protein expression. This analysis uniformly revealed a marked increase in ELOB protein levels in the cancerous tissues relative to the normal adjacent tissues.

## Discussion

**Fig. 6:**
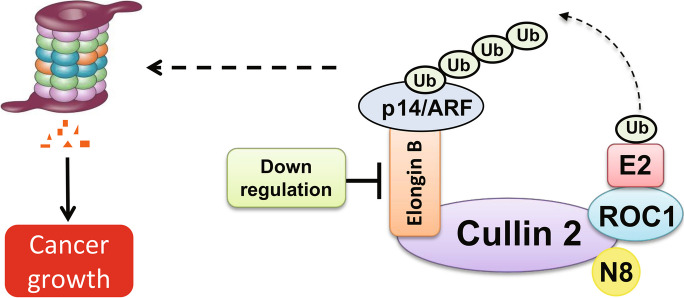
Working model of this study

Breast cancer, a leading malignancy in women globally, requires a multifaceted approach encompassing early detection, tailored treatments, and continual research to address its complex characteristics. A plethora of studies suggests that post-translational modifications of proteins are pivotal in the onset and progression of breast cancer. Among them, protein ubiquitination acts an essential role in protein degradation and is intricately related to breast cancer. Ubiquitination is a cellular process where ubiquitin molecules, with the assistance of a set of special enzymes, tag proteins within the cell, enabling the selection and specific modification of target proteins specifically. Key enzymes involved in this process encompass ubiquitin-activating enzymes, conjugating enzymes, linker enzymes, and degrading enzymes, each playing a distinct role in the intricate ubiquitination pathway. In addition, it has emerged as a novel therapeutic target for drug research and development. In this study, ELOB was discovered for the first time and it can not only act as a component of the E3 ubiquitin ligase, but also influences the biological behavior of breast cancer. Mechanistically, ELOB targets the tumor suppressor protein p14/AKF, leading to the down-regulation of p14/AKF and ultimately promoting the progression of breast cancer (Fig. 6).

ELOB acts as a part of the E3 ubiquitin ligase and mediates substrate ubiquitination by linking Cullins to substrate recognition proteins (Guo et al. 2014). ELOB is mainly involved in constituting CLR2 and CLR5 in the E3 ubiquitin ligase (Mariani et al. 2003). It is also revealed that the expression of Cullin2, the core molecule in CLR2, could predict the prognosis and radiosensitivity of patients with glioblastoma multiforme (Zheng et al. 2021). However, the regulatory role of CLR2 in tumors remains controversial. Several studies have suggested that CLR2 could exert tumor suppressor function. CRL2-VHL complex inhibits neovascularisation and tumor growth by targeting HIF-α and ubiquitinating and degrading HIF-α (Frost et al. 2021). Although, several studies have explored the relationship between CLR2 and tumors, the role of ELOB in breast cancer remain unclear. Our study showed the bio-function of ELOB and determined the upregulated expression of ELOB, which revealed the promotional effect of ELOB in breast cancer.

Our results offer additional proof of ELOB’s involvement in assembling the E3 ubiquitin ligase complex, tasked with the binding and degradation of the tumor suppressor protein p14/ARF. The tumor suppressor function of p14/ARF has been extensively documented, highlighting its ability to modulate cell growth with or without p53 (Shen et al. 2003). In stress-free cells, p14/ARF is mainly chelated in the nucleolus. But under genotoxic stress, p14/ARF is immediately redistributed to the nucleoplasm and cytoplasm and prevention of cell cycle arrest and activation of apoptosis (Repenning et al. 2021). Besides, transcription and translation of p14/ARF are tightly regulated. In 2010, Chen et al. first proved that p14/ARF can be degraded by E3 ubiquitin ligase (Chen et al. 2010). Furthermore, Zhang et al. found that the CRL2-KLHDC3 E3 ubiquitin ligase complex can recognize and degrade p14/ARF (Zhang et al. 2022). Research in non-small cell lung cancer has demonstrated that CLR2 promotes tumor growth and resistance to targeted drugs by mediating the degradation of p14/ARF through the Kelch superfamily proteins (Liu et al. 2022). However, the contribution of p14/ARF is still ambiguous in breast cancer. Our study identified the association of ELOB with the malignant biological behavior of breast cancer. Further, ELOB degrades the downstream protein p14/ARF through ubiquitination. Some inhibitors targeting E3 ubiquitin ligase have already achieved excellent efficacy in clinical trials, such as inhibitor of apoptosis (IAP) protein inhibitors and MDM2 inhibitors. Moreover, we revealed that patients with high ELOB expression may be sensitive to ATPase inhibitor and heatshock protein (HSP) inhibitor by CMap database analysis (Supplementary Fig. 1). However, inhibitors targeting ELOB are not yet available. In the future, it is expected that drugs directed against ELOB will be designed for breast cancer therapy.

## Conclusion

To sum up, our study innovatively identified ELOB as a breast cancer oncogene overexpressed in breast cancer. In addition, ELOB is an essential component of CLR2, which degrades p14/ARF by binding to and ubiquitinating it. Therefore, ELOB is anticipated to serve as an innovative treatment option for breast cancer.

## Supplementary Information

Below is the link to the electronic supplementary material.

Supplementary file1 (PDF 111 KB)

## Data Availability

The data that support the fndings of this study are available from the corresponding author upon reasonable request.
