# Supplementary figures and images for: Elongin B promotes breast cancer progression by ubiquitinating tumor suppressor p14/ARF
Source: Cell Biol Toxicol. 2024 Apr 23;40(1):24. doi: 10.1007/s10565-024-09864-7 (PMC11039524; doi:10.1007/s10565-024-09864-7)

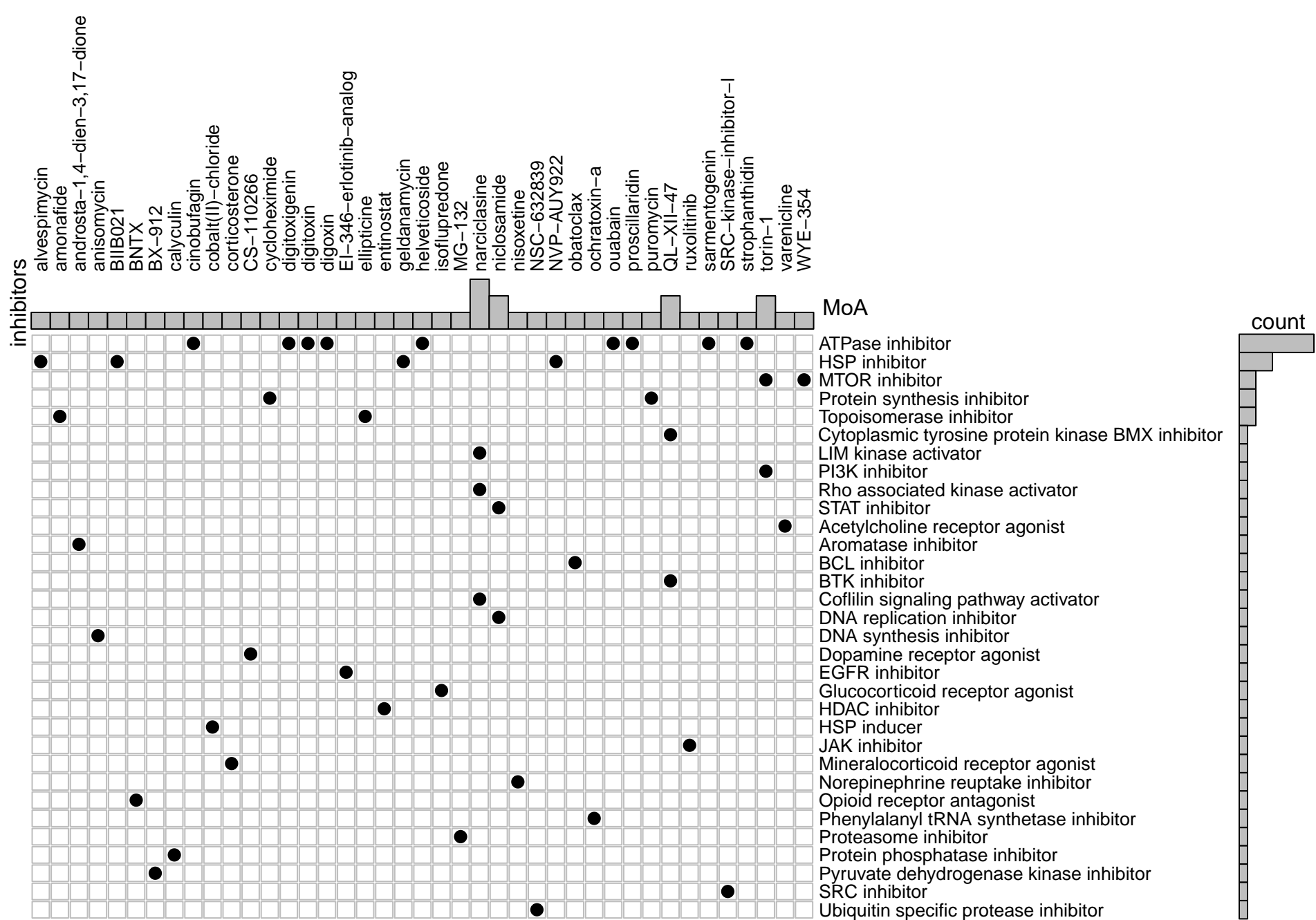

Supplement: Supplementary file 1 — Supplementary file1 (PDF 111 KB) [file 10565_2024_9864_MOESM1_ESM.pdf]
